# Bilateral Simultaneous Proximal Tibial Insufficiency Fractures in a Patient Suffering From Psoriatic Arthritis

**DOI:** 10.7759/cureus.8616

**Published:** 2020-06-14

**Authors:** Konstantinos Vlasis, Stavros Angelis, Alexandros Apostolopoulos, Dimitrios Filippou, Athanasios Papanikolaou

**Affiliations:** 1 Anatomy, National and Kapodistrian University of Athens Medical School, Athens, GRC; 2 Surgical Anatomy, National and Kapodistrian University of Athens Medical School, Athens, GRC; 3 Orthopaedics, Panagiotis and Aglaia Kyriakou Children's Hospital, Athens, GRC; 4 Orthopaedics, General Hospital Hellenic Red Cross "Korgialenio-Benakio", Athens, GRC; 5 Orthopaedics, East Surrey Hospital/Surrey and Sussex Healthcare National Health Service Trust, Redhill, GBR; 6 Surgery, National and Kapodistrian University of Athens Medical School, Athens, GRC

**Keywords:** psoriatic arthritis, insufficiency fracture, stress fracture, fatigue fracture, proximal tibia fracture, corticosteroid medication

## Abstract

Bilateral synchronous proximal tibia insufficiency fractures are rarely reported. We present a case of simultaneous proximal tibia bilateral insufficiency fractures in a 51-year-old female patient with underlying psoriatic arthritis, who was on chronic steroid medication. She reported sudden onset of bilateral knee pain after intense workout one week ago. Initial clinical and X-ray evaluation did not reveal significant pathology. Four weeks later, due to persistent pain in the absence of significant radiographic findings during follow-up, the patient was referred for MRI, which revealed fractures of both proximal tibias. A "mixed" treatment protocol was applied. In particular, this protocol included combination of rest and intermittent removable knee ranger braces immobilization with weight-bearing when applied. The patient went on to make a full recovery. Chronic inflammatory disorders accompanied by suspicious clinical manifestations should be thoroughly inspected. Diagnostic and treatment protocols should be further discussed and implemented.

## Introduction

There are two types of stress fractures [1]. The most commonly reported and well-described type is the fatigue fracture that results from irregular or prolonged repetitive stress on normal bone. These lesions are regularly seen in weight-bearing lower limb bones in athletes and military recruits with an incidence ranging from 5% to 30% [1-3]. Insufficiency fractures are also common; however, they require presence of an abnormal medical condition that affects bones (osteoporosis) by making them more vulnerable to load application [1]. Subsequently, poor bone quality diminishes energy load required to produce breakage [1,4].

When referring to insufficiency fractures most physicians relate these injuries with vertebrae, proximal femur and sacrum lesions [1]. Proximal tibia insufficiency fractures are seldomly reported and treatment protocols usually involve rest and weight-bearing constraint measures [3,5,6]. Misdiagnosis leading to inappropriate management is typical for this condition since radiographic evaluation in early stages is usually inconclusive [3-6].

We report a case of simultaneous proximal tibia bilateral insufficiency fractures in a 51-year-old female patient with underlying psoriatic arthritis. To the best of our knowledge, only one similar case has been reported in literature; however, this patient suffered psoriasis and bilateral proximal tibia insufficiency fractures and was under methotrexate and cortisone medication [7]. Our patient was only on corticosteroids.

## Case presentation

A 51-year-old female Caucasian patient attended the Outpatient Orthopaedic Clinic of ''Korgialenio-Benakio'' Hellenic Red Cross Hospital, due to sudden onset of bilateral knee pain after vigorous exercise (treadmill workout), one week ago. She could not recall any recent history of trauma. Medical history of psoriatic arthritis was recorded. She had been under cortisone medication for the past four years. No other medical or surgical history was reported.

As far as relevant past interventions are concerned, she reported that she did not seek immediate medical attention prior to her visit to the Outpatient Clinic, even though pain was extremely intense and not consistent with usual musculoskeletal discomfort she had experienced in the past, related to her medical condition (psoriatic arthritis). She reported utilization of non-steroidal anti-inflammatory drugs (NSAIDs), cryotherapy and rest, with subsequent improvement. However, she stated excruciating pain in standing position and especially after a few steps, forcing her to reduce walking distance.

**Figure 1:**
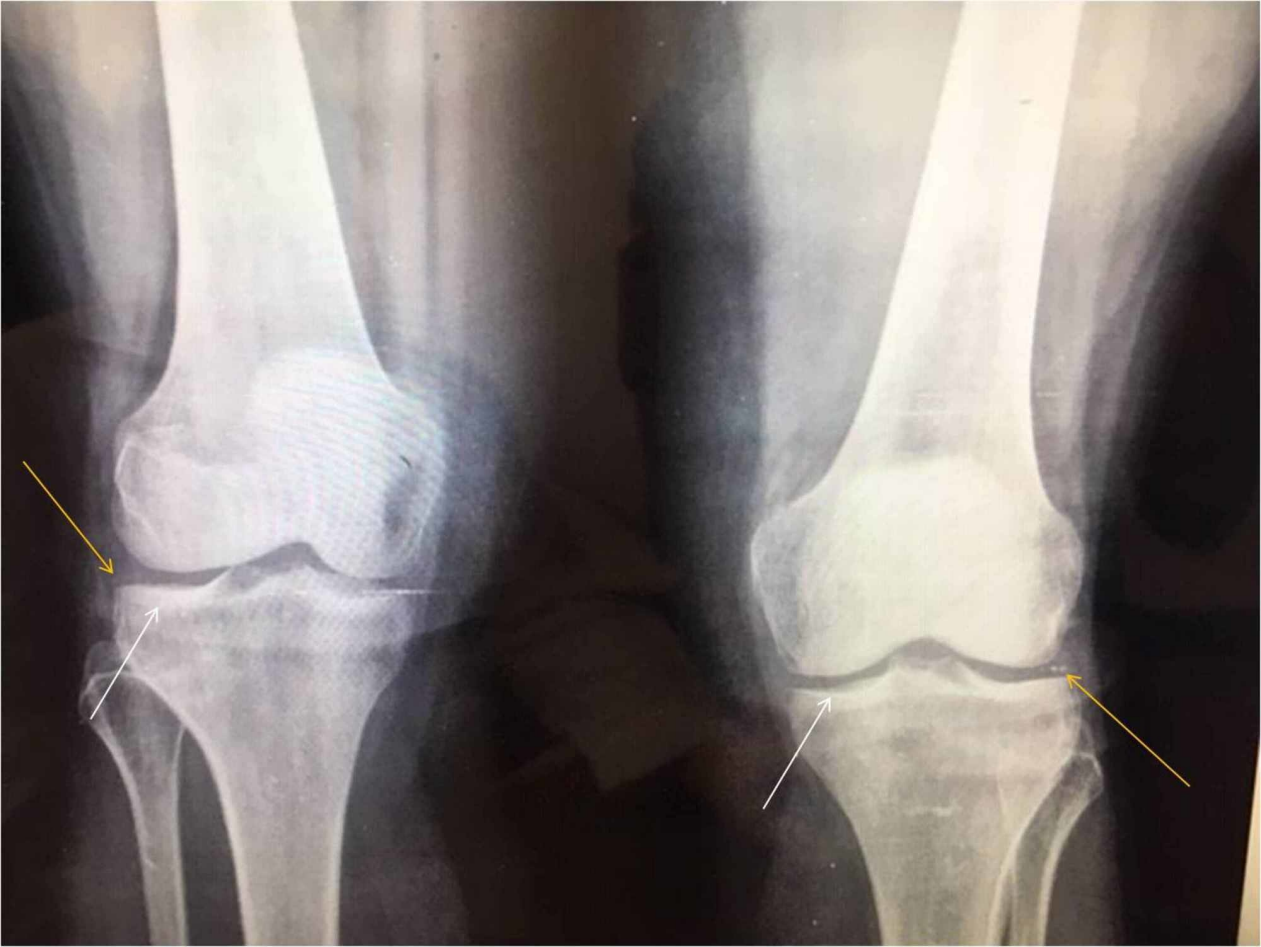
Anteroposterior X-ray views of both knees. Mild degenerative lesions with no signs of significant pathology. The white arrows point to eburnation of bone and the yellow arrows point to osteophytes and meniscal calcification.

During physical examination, the patient was ambulatory and able to bear weight. No restriction in range of motion (ROM), instability or locking sensation of both knees was recorded. No joint effusion was obvious; however, mild diffuse swelling and pain during palpation over both medial proximal tibia regions were noted. Anteroposterior (AP) and lateral (L) X-ray views did not reveal significant pathology other than mild degenerative lesions (Figure 1).

**Figure 2:**
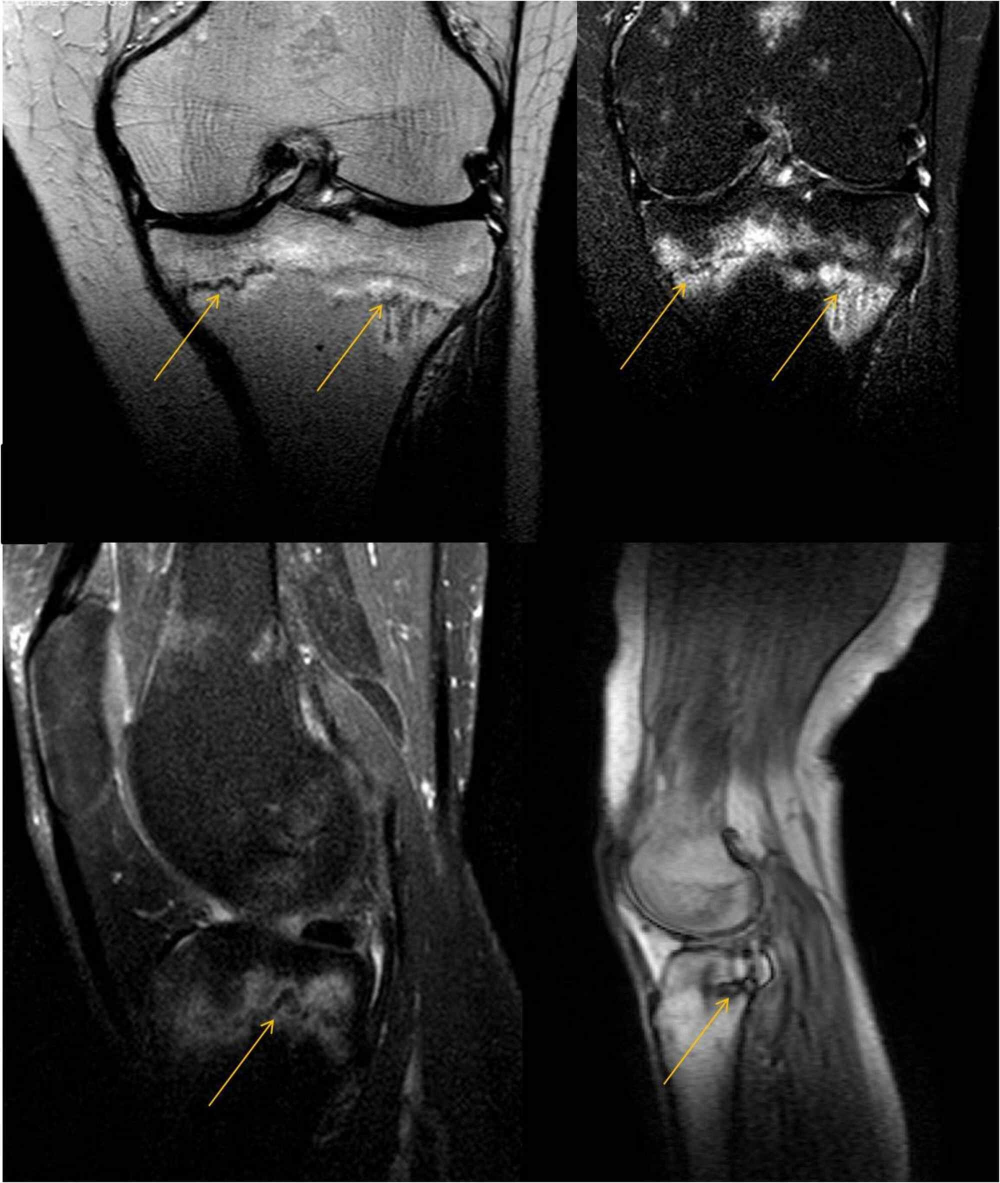
MRI of the left knee. The yellow arrows point to insufficiency fracture lines. Edema is obvious around the fracture lines.

NSAIDs were prescribed, and the patient was advised to continue cold therapy and avoid weight-bearing. Persistence of pain with no significant radiographic pathology at follow-up, four weeks later, led to MRI referral for the left knee where pain was reported to be more severe. MRI revealed stress/fatigue/insufficiency fracture of the proximal tibia (Figure 2).

**Figure 3:**
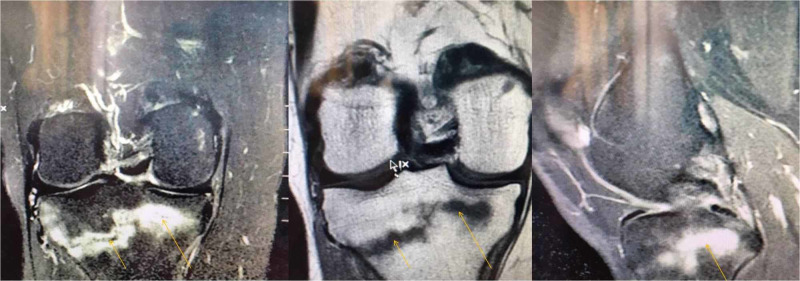
MRI of the right knee. The yellow arrows point to insufficiency fracture lines. Edema is obvious around the fracture lines.

Suspicion of similar pathology in the right proximal tibia was confirmed with another MRI carried out two days later (Figure 3). Basic laboratory data were within normal range. 

A protocol of conservative measures was applied. This included rest and intermittent removable knee ranger braces immobilization. Non-load bearing was employed when braces were removed, and weight-bearing, as tolerated, with walking aids was utilized when braces were applied. Active and passive mobilization exercises of both knees were applied during rest hours.

**Figure 4:**
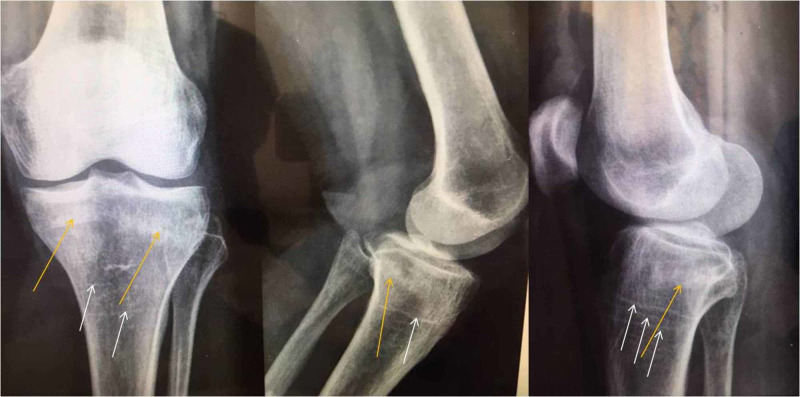
X-rays with callus formation (yellow arrows) implying fracture healing. Also, note the growth arrest or Harris lines (white arrows) that usually result from pathologic levels of stress during bone development.

The patient reported direct analgesic response to the protocol from the first follow-up meeting two weeks after initiation. Six weeks post-initiation of the protocol, the patient was encouraged to remove the casts. At eight weeks, she reported no pain or discomfort, examination revealed no limitation of ROM or muscle mass loss and X-ray callus formation was visible (Figure 4). One year post-protocol initiation, no signs of tibial plateaus collapse were noted.

## Discussion

Insufficiency fractures are also known as osteoporotic, fragility, pathological, age-related or minimal-trauma fractures [8]. Risk factors are related to osteoporotic conditions that may impair bone strength. Subsequently, osteoporosis may be related to ageing, female sex and early menopause, white race, low body mass index (BMI), sedentary life and prolonged immobilization, chronic inflammatory disorders, corticosteroid and methotrexate medication, smoking and alcohol abuse [1,4,8,9]. Our patient mustered a series of these factors; the most important of which were that she suffered from psoriatic arthritis and was on chronic steroid use. Furthermore and besides female gender, she was a white Caucasian with low BMI (18.95) who lead a sedentary life until she decided to embark on a workout programme.

Psoriatic arthritis has been associated with several comorbid health conditions. As far as osteoporosis and insufficiency fractures are concerned, controversial results have been reported [10]. However, large pooled analysis studies support the relation between this inflammatory arthropathy and pathological fractures [10]. In particular, pathogenesis is related to decreased bone mineral density, low vitamin D, chronic inflammation, high rates of smoking, and corticosteroid and methotrexate medication [4,10]. Reduced bone mineral density is associated with intensified bone resorption subsequent to increased concentrations of tumour necrosis factor-a (TNF-a) and interleukin 6 (IL-6) [11]. The principal result of cortisone treatment on bone is impaired collagen synthesis. Furthermore, glucocorticoids decrease calcium gastrointestinal absorption [12].

The main symptom of insufficiency fracture is related to the spontaneous onset of severe pain in the affected area [8]. Pain is aggravated via activity and alleviated by rest [7]. In some cases, discomfort may be noted during activity, developing into persistent pain at rest [9]. Moreover, tenderness and effusion localized over the adjacent bone instead of the joint, as well as functional impairment is usually noted [8,13]. Commonly, there is no history of preceding trauma and examination may suggest an inflammatory process [8]. Most of these manifestations were recorded in our case, directing initial diagnosis towards mild degenerative lesions. Furthermore, our initial diagnosis was supported by X-ray evidence. However, stress fractures are well known for appearance without obvious breakage or lucency lines, especially in early stages and around the knee [4,7,9,14]. In particular, diagnostic sensitivity is less than 15% in initial X-rays; moreover, 50% of stress fractures are missed at first follow-up [9,14]. Having this in mind during the initial examination, we recommended non-weight-bearing measures and follow-up in a month. Obviously, sensitivity depends on time elapsed between initial injury and X-ray depiction [14]. Yet, during follow-up, no significant radiographic pathology was detected. Further imaging investigation was decided. Nowadays, there is a variety of imaging techniques that can be selected, such as bone scans, CT scans, or MRI scans [4,7,8,14]. MRI was selected for two reasons in our case. First of all, MRI has the benefit to evaluate both skeletal structures and soft tissues in the knee [7]. Secondly, it is supported in more recent literature for accurate diagnosis of the common transverse or oblique tibial stress fractures [14].

Initial treatment options for proximal tibia stress fractures usually involve conservative measures; however, in case of complications, surgical treatment may be considered [7,8]. Non-surgical measures principally rely on a complete or partial non-weight-bearing period [7]. Immobilization in either a cast or a brace is the main means to achieve this. Decreased activity can often lead to healing of stress fractures. However, a consideration that physicians should keep in mind is that immobilization may also lead to adverse effects like malunions and non-unions; varus failure of the tibial plateau may be the reason for malalignment that sequentially leads to pain [7]. Additionally, some studies support that patients suffering psoriasis are more likely to live sedentary lives and avoid physical activities [10]. This could predispose them to insufficiency fractures; furthermore, it could predispose in favour of the complications previously described [10]. These patients should be encouraged to utilize more weight-bearing physical activities in a controlled manner, as this might improve bone regeneration and remodelling. [10]. Therefore, we have applied a "mixed" treatment protocol. This included rest and intermittent removable knee ranger braces immobilization with weight-bearing when applied. Our patient demonstrated no muscle mass loss, no stiffness of her knees and no tibial plateau collapse, and callus formation was visible two months post-treatment protocol was initiated.

## Conclusions

Bilateral synchronous proximal tibia insufficiency fractures have not been frequently reported. We have described this injury in a patient suffering psoriatic arthritis and treated with cortisone medication. Chronic inflammatory disorders accompanied by suspicious clinical manifestations should be thoroughly inspected. Diagnostic and treatment protocols should be further discussed and implemented.
